# Age-disparate and intergenerational sex partnerships and HIV: the role of gender norms among adolescent girls and young women in Malawi

**DOI:** 10.1186/s12889-024-17868-5

**Published:** 2024-02-22

**Authors:** Domonique M. Reed, Elizabeth Radin, Evelyn Kim, Nellie Wadonda-Kabondo, Danielle Payne, Myrline Gillot, Andreas Jahn, George Bello, Thokozani Kalua, Jessica E. Justman

**Affiliations:** 1 Department of Epidemiology, Mailman School of Public Health, Columbia University, 722 W 168th St, Floor 7, New York, NY USA; 2 Mailman School of Public Health, ICAP at Columbia University, Columbia University, New York, NY USA; 3 U.S. Centers for Disease Control and Prevention, Lilongwe, Malawi; 4 Total Solutions, Inc, New York, NY USA; 5 Department of HIV and AIDS, Ministry of Health, Lilongwe, Malawi; 6 Department of Global Health, International Training and Education Center for Health, University of Washington, Seattle, WA USA

**Keywords:** Age-mixing sex partnership, Gender norms, Adolescent girls and young women, HIV, Malawi

## Abstract

**Background:**

Age-mixing (age-disparate [5–9 years difference] and intergenerational [≥ 10 years difference]) partnerships are hypothesized drivers of HIV in adolescent girls and young women (AGYW; 15–24 years). These partnerships are often associated with increased gender inequities which undermine women’s agency and assertiveness. We assessed whether age-mixing partnerships were associated with HIV in Malawi and if endorsement of inequitable gender norms modifies this relationship.

**Methods:**

We analyzed data from the Malawi Population-based HIV Impact Assessment, a nationally representative household survey conducted in 2015–2016. Participants underwent HIV testing and completed questionnaires related to actively endorsed gender norms and sexual risk behavior. We used multivariate logistic regression and multiplicative interaction to assess associations among AGYW who reported the age of their primary sex partner from the last year.

**Results:**

The analysis included 1,958 AGYW (mean age = 19.9 years, SD = 0.1), 459 (23.4%) and 131 (6.7%) of whom reported age-disparate and intergenerational partnerships, respectively. AGYW in age-mixing partnerships accounted for 13% of all AGYW and were older, more likely to reside in urban areas, to be married or cohabitating with a partner, and to have engaged in riskier sexual behavior compared with AGYW in age-concordant partnerships (*p* < 0.05). HIV prevalence among AGYW in age-disparate and intergenerational partnerships was 6.1% and 11.9%, respectively, compared with 3.2% in age-concordant partnerships (*p* < 0.001). After adjusting for residence, age, education, employment, wealth quintile, and ever been married or cohabitated as married, AGYW in age-disparate and intergenerational partnerships had 1.9 (95% CI: 1.1–3.5) and 3.4 (95% CI: 1.6–7.2) greater odds of HIV, respectively, compared with AGYW in age-concordant partnerships. Among the 614 (31% of the study group) who endorsed inequitable gender norms, AGYW in age-disparate and intergenerational partnerships had 3.5 (95% CI: 1.1–11.8) and 6.4 (95% CI: 1.5–27.8) greater odds of HIV, respectively, compared with AGYW in age-concordant partnerships.

**Conclusions:**

In this Malawi general population survey, age-mixing partnerships were associated with increased odds of HIV among AGYW. These findings highlight inequitable gender norms as a potential focus for HIV prevention and could inform interventions targeting structural, cultural, and social constraints of this key group.

**Supplementary Information:**

The online version contains supplementary material available at 10.1186/s12889-024-17868-5.

## Introduction

Despite substantial progress in Human Immunodeficiency Virus (HIV) prevention research, adolescent girls and young women (AGYW), aged 15 to 24 years, remain a particularly vulnerable population in sub-Saharan Africa. In 2017, the Joint United Nations Program on HIV/AIDS (UNAIDS) reported that AGYW represent 10% of the total sub-Saharan African population but account for 25% of all new HIV cases globally [1, 2]. Similar to AGYW in many other sub-Saharan African countries, AGYW in Malawi are twice as likely to be living with HIV compared to males of the same age group [2, 3]. One postulated driver of infections in AGYW is age-mixing sexual partnerships, characterized as age-disparate (≥ 5 years age difference) and intergenerational (≥ 10 years age difference) partnerships [4, 5]. 

The Socioecological Model holds that features of the social and structural environment enable and constrain individual behavior and attitudes; therefore, there are multiple factors beyond individual characteristics that likely perpetuate the HIV disparity observed among AGYW who engage in age-mixing partnerships [6, 7]. Potential pathways for increased HIV risk in age-mixed partnerships include the higher prevalence of HIV among older men and potential power imbalances leading to riskier sexual practices [8, 9]. HIV prevalence is almost five times higher in men 25 years or older compared with adolescent boys and young men (15–24 years) in some regions of sub-Saharan Africa, and overall, there is lower uptake of HIV treatment among men of all ages compared with women [3, 8, 10–13]. Age-disparate and intergenerational relationships are also associated with gender inequities and power imbalances due to the women’s socioeconomic dependency on their partners and may result in increased risk of male-perpetuated intimate partner violence [14, 15]. Such asymmetries make it difficult for AGYW to exercise agency over their health and sexual behavior. As a result, age-mixed partnerships have been associated with riskier sexual behavior, such as condomless sex, transactional sex, and male partners having concurrent sexual partnerships with other women, increasing AGYW’s risk of HIV [16]. 

Although the hypothesized relationship between age-mixing sex partnerships and increased HIV risk seems plausible, the research remains equivocal. Evidence from cross-sectional and ecological studies suggest that age-disparate sex partnerships contribute to the high risk of HIV among AGYW, while longitudinal studies have been inconsistent [14, 16–20]. Inconsistent findings may reflect variations in HIV risk in some communities compared with overall national HIV risk, including differences in risk between urban and rural areas; for example, many of the longitudinal studies were conducted in rural settings [18, 20, 21]. Additionally, differences in study populations, resource accessibility, and other country-specific contextual factors may explain the inconsistencies, such as HIV-specific program priorities, laws, and cultural norms [19, 20, 22–26]. Although the association between age-mixing patterns and HIV have been assessed in many countries in Sub-Saharan Africa, such as South Africa, Kenya, Zimbabwe, and Uganda, few studies have assessed the association in Malawi [19, 20, 22–28]. One of the two studies conducted in Malawi, used a dyadic analysis and found consistent underestimation of partner age by AGYW. Although the relationship between age-mixing and HIV was not assessed, these findings highlight the important consequence of this underestimation which could lead to misjudging the extent and impact of age-mixing partnership. The other study did assess the relationship between age-mixing and HIV and found a slight increased risk of HIV with older partners (2 + years) but beyond 12 years older the relationship became protective [10, 29]. Both studies used the same cohort which was conducted in Likoma Island, Malawi.

Malawi is well-suited for an analysis of age-mixing partnerships and HIV. The 2015 Malawi Demographic and Health Survey reported that 42% of young women were married before age 18 compared with just 6% in South Africa [30–32]. Despite Malawi Parliament passing a law in 2017 that banned marriage before the age of 18, enforcement has not been consistent in all jurisdictions [32]. The younger average marriage age in Malawi may further fuel or be a symptom of gender inequality that removes personal agency related to financial expenditures, personal relationships, violence, and bargaining power within marriages [33]. These gender inequalities can increase AGYW’s vulnerability to HIV [34–36]. Unfortunately, male superiority is frequently embedded in cultural and gender norms, leading to a paucity of quantitative research assessing the role of inequitable gender norms and HIV among AGYW in Malawi [28]. Given this context, further complicated by Malawi’s generalized HIV epidemic and the impending demographic youth bulge, the number of AGYW potentially at risk for HIV transmission through age-mixing partnerships could be rising [37]. 

The 2016 Malawi Population-based HIV Impact Assessment (MPHIA) was a nationally representative, HIV-focused cross-sectional survey of the general population, and the resulting data provided a unique opportunity to compare the associations between age-disparate and intergenerational sex partnerships and HIV with age-concordant sex partnerships, and to assess whether the strength of this association varies by endorsement of inequitable gender norms among a population-based sample of AGYW. We hypothesized that endorsement of inequitable gender norms would significantly amplify the association between HIV and age-mixing patterns among AGYW. The findings from this study will be one of the first studies to quantify the modifying role of inequitable gender norms on age-mixing sexual partnerships and HIV among AGYW in Malawi.

## Methods

### Study population, sample, and ethics

MPHIA, conducted between November 2015 and August 2016, used a two-stage, stratified cluster sampling design to select a nationally and zonally representative sample. The resulting sample included 14,268 households across 500 enumeration areas [38]. During household visits, consenting heads of households and emancipated minors (< 18 years who are married or free from any legally competent representative as defined by Malawi law) completed a household questionnaire and provided a roster of all the members within the household. Survey staff then conducted private face-to-face interviews with consenting participants and collected information on demographics, behaviors, and beliefs related to gender norms, sexual risk behavior, and partner status. For children aged 15 to 17 years, a guardian or parent provided permission for interviewers to approach them and obtain assent [39]. 

This analysis included AGYW who reported the age of their primary sexual partner (“How old is your [partner]?”) in the last 12 months. For this analysis, primary sexual partner is defined as the most recent sexual partner, as the most recent partner reduces recall bias and evidence suggests that sexual behaviors are consistent across partnerships [40]. Additionally, the majority of AGYW included in this analysis reported a single partner. This analysis excluded participants who did not report having a primary sexual partner in the previous 12 months, did not know their primary partner’s age, or for whom there was missing data on partner’s age or key HIV indicators.

Written informed consent or assent was documented via electronic signature, with witnesses verifying consent for illiterate individuals. Informed consent procedures with illiterate individuals involved the use of an impartial witness, selected by the potential participant, who also signed or marked on the consent form on the tablet. If an impartial witness could not be identified, the potential participant was deemed ineligible.

The Centers for Disease Control and Prevention Institutional Review Board (IRB), the Columbia University Irving Medical Center IRB, and National Health Science Research Committee of Malawi approved the protocol for MPHIA.

### Measures

Using UNAIDS and World Health Organization definitions, we categorized AGYW as being in an age-disparate sex partnership if their partner was ≥ 5 years older than them or being in an intergenerational sex partnership if their partner was ≥ 10 years older [41]. Our reference group was AGYW in an age-concordant sex partnership if their reported sexual partner was < 5 years older or younger than them [41]. Our primary outcome was serologically confirmed HIV status, as previously described [42]. Briefly, HIV status was ascertained using Determine HIV-1/2 Rapid Test (Abbott Molecular Inc., Des Plaines, Illinois, United States) and then reactive samples were confirmed with Uni-Gold™ (Trinity Biotech, plc. Wicklow, Ireland). Recent infection status was defined by an algorithm based on HIV-1 Limiting Antigen (LAg)-Avidity enzyme immunoassay (Sedia Biosciences, Portland, OR, USA), HIV-1 RNA (viral load), and antiretroviral status (self-reported use or detectable ARVs) [42]. 

Our hypothesized effect modifier was endorsement of inequitable gender norms. The MPHIA questionnaire assessed inequitable gender norms through a modified (i.e., reduced number of items, adapted to include responses relevant for women) Gender Equitable Men (GEM) Scale. This nine item scale measures attitudes toward gender norms in intimate partnerships or differing social expectation for men and women: (1) “Who usually makes decision about health care for yourself?” (I do/ Spouse or Partner/We both do/ Someone else); (2) “Who generally decides about how the money you receive is spent?” (I do/ Spouse or Partner/We both do/ Someone else); (3) “Do you believe it is right for a man to hit or beat his wife/partner if she goes out without telling him?” (Yes/No); (4) “Do you believe it is right for a man to hit or beat his wife/partner if she does not take care of the children?” (Yes/No); (5) “Do you believe it is right for a man to hit or beat his wife/partner if she argues with him?” (Yes/No); (6) “Do you believe it is right for a man to hit or beat his wife/ partner if she refuses to have sex with him?” (Yes/No); (7) “Do you believe it is right for a man to have sex with other women if his wife/partner refuses to have sex with him?” (Yes/No); (8) “Do you believe a person should tolerate violence to keep the family together?” (Yes/No); and (9) “Do you believe women who carry condoms have sex with a lot of men?” (Yes/No). We categorized participants who responded, “Spouse or partner”, “We both do”, “Someone else”, or “Yes” to any of the items above as endorsing inequitable gender norms [43]. AGYW who affirmed any of the nine items were categorized as endorsing inequitable gender norms. We selected to include “We both do” as an affirmative response due to considerations of autonomy and agency regardless of relationship status (i.e., marital status).

We analyzed covariates associated with HIV infection in AGYW’s participation in age-disparate and intergenerational sex partnerships and assessed whether those covariates mediated or confounded the association between these sexual relationships and HIV based on the literature [16, 17, 39]. The final model included the following confounders: age, residence, education, employment in the last 12 months or student status, household wealth quintile, and ever been married or cohabitated. Sexual behaviors, such as condom use and HIV status of partner were considered mediators between age-mixing partnerships and HIV; thus, we did not control for their effects to assess the total average association [44]. 

### Statistical analysis

This analysis used data weighted to account for selection probability, nonresponse, and noncoverage [38]. SAS Version 9.4 (SAS, Cary, NC) were used for data cleaning and analyses. We used jackknife replicate weights for variance estimation and presented weighted percentages. To compare demographic and behavioral characteristics among AGYW in age-disparate sex partnerships versus AGYW in age-concordant sex partnerships, we used Rao-Scott chi-square since we are using survey data. To assess the association between age-disparate sex partnership status and HIV, logistic regression models with robust standard errors were used to estimate the unadjusted and adjusted odds ratio (OR) and 95% CI. To assess the effect of inequitable gender norms, cross-multiplicative terms were included into the unadjusted and adjusted models. Significance level was set to an alpha of 0.05.

## Results

**Fig. 1:**
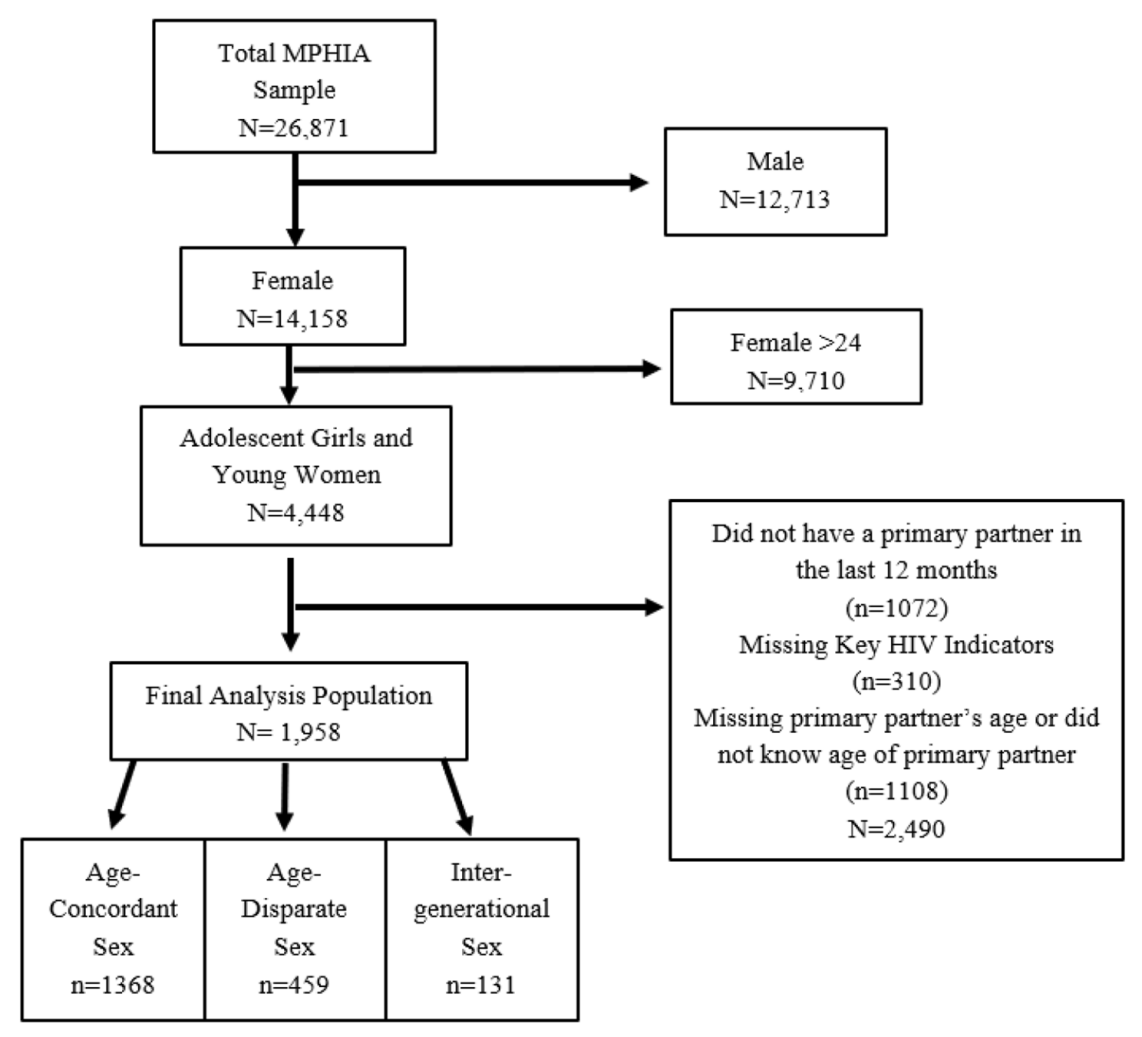
Flow Diagram for Inclusion of Study Participants

A total of 26,871 children and adults took part in MPHIA, of whom 4,448 were AGYW (Fig. 1). Of these, 1,072/4,448 (24%) did not report having a primary partner in the past 12 months, 1,108/4448 (25%) had missing information on primary partner’s age and 310 (7%) were missing HIV indicator data. The remaining 1,958/4448 (44%) met the inclusion criteria for this analysis, of whom 671 (42.3%) were adolescent girls and 1,287 (57.7%) were young women.

Of the 1,958 (Table 1) in the study group, (i.e., reporting a primary partnership in past 12 months and reporting the age of the partner), 459 (23.4%) reported being in an age-disparate sex partnership, 131 (6.7%) reported being in an intergenerational sex partnership and 1,368 (69.9%) were in an age-concordant sex partnership. Among the entire 4,448 AGYW, 590 (13%) reported being in age-disparate or age-intergenerational relationships. Compared with AGYW included in the sample, those AGYW without a primary partner were younger (15–19 years), more likely to reside in a household with a higher wealth quintile and less likely to be married or cohabitating; and those AGYW who did not know the age of their partner were less likely to be employed and less likely to ever engage in transactional sex (*p*-values < 0.001).

**Table 1:** Characteristics of Adolescent Girls and Young Women in Age-Disparate Sex Partnerships compared to Age-Concordant Sex Partnerships

Characteristics		Overall *n* = 1958	Age-Concordant Sex *n* = 1368	Age-Disparate Sex *n* = 459	Intergenerational Sex *n* = 131	*p*-value
Age						< 0.0001
	15–19	671 (42.3%)	523 (46.9%)	122 (30.4%)	26 (29.0%)	
	20–24	1287 (57.7%)	845 (53.1%)	337 (69.6%)	105 (71.0%)	
Residence						< 0.0001
	Urban	804 (21.4%)	527 (19.0%)	213 (28.5%)	64 (25.9%)	
	Rural	1154 (78.6%)	841 (81.0%)	246 (71.5%)	67 (74.1%)	
Education						0.0387
	No Education	80 (5.0%)	51 (4.5%)	19 (4.7%)	10 (12.3%)	
	Primary	1136 (64.5%)	791 (64.7%)	268 (64.5%)	77 (61.7%)	
	Secondary or More	742 (30.5%)	526 (30.8%)	172 (30.8%)	44 (26.0%)	
Employed in the last 12 months or enrolled in school						< 0.0001
	Yes	641 (33.5%)	497 (37.2%)	112 (22.8%)	32 (26.2%)	
	No	1249 (66.5%)	829 (62.8%)	330 (77.2%)	90 (73.8%)	
Household Wealth Quintile						0.0172
	Lowest	255 (16.7%)	190 (17.9%)	53 (14.7%)	12 (12.2%)	
	Second	280 (18.1%)	213 (19.2%)	50 (13.1%)	17 (21.3%)	
	Middle	319 (20.9%)	234 (21.1%)	73 (22.4%)	12 (12.9%)	
	Fourth	395 (21.0%)	270 (20.3%)	93 (21.8%)	32 (26.8%)	
	Highest	709 (23.2%)	461 (21.5%)	190 (28.0%)	58 (26.8%)	
Ever Married or lived together as if married						< 0.0001
	Yes	1373 (68.5%)	875 (62.8%)	378 (82.8%)	120 (86.6%)	
	No	585 (31.5%)	493 (37.2%)	81 (17.2%)	11 (13.4%)	
Multiple Partners in past 12 months						0.3377
	1 Partner	1821 (93.9%)	1272 (93.8%)	427 (95.0%)	122 (90.5%)	
	2 + Partners	137 (6.1%)	96 (6.2%)	32 (5.0%)	9 (9.5%)	
Ever had anal sex						0.0930
	Yes	51 (2.4%)	28 (2.0%)	17 (3.8%)	6 (3.4%)	
	No	1891 (97.6%)	1329 (98.0%)	437 (96.2%)	125 (96.6%)	
Condom use at last sex						< 0.0001
	Yes	531 (27.5%)	425 (31.4%)	88 (17.9%)	18 (14.2%)	
	No	1426 (72.5%)	943 (68.6%)	370 (82.1%)	113 (85.8%)	
Alcohol use at last sex						0.0876
	AGYW and/or partner drinking	150 (6.7%)	87 (6.0%)	49 (9.0%)	14 (7.7%)	
	Neither drinking	1800 (93.3%)	1277 (94.0%)	406 (91.0%)	117 (92.2%)	
HIV status of partner						0.0003
	Positive	43 (1.8%)	22 (1.2%)	11 (2.4%)	10 (7.4%)	
	Negative	1187 (58.3%)	835 (58.3%)	276 (60.9%)	76 (49.8%)	
	Don’t Know	726 (39.9%)	509 (40.5%)	172 (36.7%)	45 (42.8%)	
Ever sold sex for money						0.3800
	Yes	107 (6.4%)	76 (6.6%)	21 (5.1%)	10 (9.1%)	
	No	1851 (93.6%)	1292 (93.4%)	438 (94.9%)	121 (90.9%)	
HIV Status						< 0.0001
	Positive	115 (4.3%)	59 (3.2%)	34 (6.1%)	22 (11.9%)	
	Negative	1843 (95.7%)	1309 (96.8%)	425 (93.9%)	109 (88.1%)	
Endorsement of one or more Inequitable Gender Norms						0.4393
	Yes	614 (33.1%)	416 (32.6%)	151 (33.1%)	47 (39.5%)	
	No	1343 (66.9%)	952 (67.4%)	308 (66.9%)	83 (60.5%)	

Missing: Endorsement of Inequitable Gender Norms, Condom use at last sex, and alcohol use at last sex, HIV status of partner (< 1%); Ever had anal sex (0.8%); Employed in the last 12 months or enrolled in school (3%)

Mean age of the study group was 19.9 (SD = 0.1). Members of age-disparate and intergenerational sex partnerships were slightly older (*p* < 0.001), and a greater proportion lived in an urban area of Malawi (*p* < 0.001), had lower educational attainment (*p* = 0.0387), were unemployed or not in school (*p* < 0.0001), and had a higher household wealth quintile (*p* = 0.0172) compared with those in age-concordant sex partnerships. As related to partnership dynamics and sexual behaviors, a greater proportion of members in age-disparate and intergenerational sex partnerships had been or were currently married or cohabitating as if married (*p* < 0.001), reported not using a condom at last sex with primary partner in the last 12 months (*p* < 0.001), and had a partner living with HIV (*p* = 0.003). The prevalence of HIV was 6.1% among AGYW in age-disparate sex partnerships, 11.9% among AGYW in intergenerational sex partnerships, and 3.2% among AGYW in age-concordant sex partnerships (*p* < 0.001). Among those that were living with HIV, 54.4% were aware of their HIV status (i.e., self-reported aware or had detectable levels of ARV), and of those, 49.2% were in age-concordant relationships, 28.6% were in age-disparate relationships, and 22.2% were in intergenerational relationships. In MPHIA, there were 6 recent infections among all AGYW (unweighted; 3 in age-disparate sex partnerships and 3 in age-concordant sex partnerships).

Among the 1,958 in a primary partnership, 614 (31%) AGYW endorsed inequitable gender norms (Supplemental Material Fig. 1); of these, almost half (46.7%) endorsed at least two of the norms. Although differences by relationship type across all items were non-significant, a substantial proportion of participants within each group endorsed the norms: husband or someone else makes healthcare decisions for them (16.8%); believe a person should tolerate violence to keep the family together (12.7%); and believe that women who carry condoms have sex with a lot of men (35.9%).

Table 2 displays the models assessing the associations of interest. After adjusting for residence, age, education, employment, wealth quintile, and marital/cohabitation status, AGYW in age-disparate partnerships had a 1.9 (95% CI: 1.1–3.5) higher adjusted odds of HIV compared with AGYW in age-concordant sex partnerships. AGYW in intergenerational sex partnerships had a 3.4 (95% CI: 1.6–7.2) higher adjusted odds of HIV compared with AGYW in age-concordant sex partnerships.

**Table 2:** Unadjusted and Adjusted Odds Ratios and 95% Confidence Intervals for HIV Infection by Partnership Status and Assessment of the Effect Modification by Endorsement of Inequitable Gender Norms

		Unadjusted	Adjusted^1^		
		OR	95% CI	OR	95% CI
Overall					
	Age-Disparate Sex with Partner ≥ 5 years older	2.0	1.1–3.4*	1.9	1.1–3.5*
	Intergenerational Sex with Partner ≥ 10 years older	4.1	2.0-8.2*	3.4	1.6–7.2*
Endorsement of one or more Inequitable Gender Norms					
	Age-Disparate Sex with Partner ≥ 5 years older	3.1	1.1–9.5*	3.5	1.1–11.8*
	Intergenerational Sex with Partner ≥ 10 years older	4.4	1.1–17.6*	6.4	1.5–27.8*
Non-endorsement of Inequitable Gender Norms					
	Age-Disparate Sex with Partner ≥ 5 years older	0.9	0.73–2.6	1.1	0.7–1.5
	Intergenerational Sex with Partner ≥ 10 years older	1.8	0.8-3.0	1.1	0.7–1.8

^1^ Adjusted for residence, age, education, employment, wealth quintile, and ever been married or cohabitated as married. Reference category is Age-Concordant Sex Partner.

^*^p < 0.05

When assessing the endorsement of inequitable gender norms as a modifier, after adjusting for the confounders listed previously, we found that among AGYW who endorsed one or more inequitable gender norm, members of age-disparate sex partnerships had a 3.5 (95% CI: 1.1–11.8) greater adjusted odds of HIV compared with members of age-concordant sex partnerships. Also, among AGYW who endorsed inequitable gender norms, members of intergenerational sex partnerships had a 6.4 (95% CI: 1.5–27.8) greater adjusted odds of HIV compared with members of age-concordant sex partnerships.

## Discussion

In this nationally representative, HIV-focused survey conducted in Malawi, almost one-third (30.1%) of AGYW in a primary sex partnership in the prior 12 months were in age-disparate or intergenerational sex partnerships. This group, accounting for 13% of all AGYW included in this nationally representative analysis, had at least a two-fold higher HIV prevalence compared with AGYW in age-concordant sex partnerships. In addition, one-third of AGYW in all groups endorsed beliefs related to male partner’s agency over healthcare decisions for women, tolerance of violence to maintain a family, and presumed sexual partner concurrency of women who carry condoms. Overall, these findings support our hypothesis that the strength of the association between HIV status and age-mixing partnerships persists among those endorsing inequitable gender norms, particularly among intergenerational relationships.

Several studies have shown that young women with older partners are more likely to engage in higher risk sexual behavior such as condomless sex and having a partner who is living with HIV, which is consistent with findings from our study [20, 22–25]. The majority of AGYW in age-mixing partnerships in this study were married or cohabitating, which we found was associated with engaging in this higher risk behaviors [11, 45]. Riskier sexual behavior could serve as a mediator between being married or cohabiting and HIV among AGYW in age-mixing partnerships and should be explored further. In response to the high and sustained HIV incidence among AGYW, PEPFAR created the Determined, Resilient, Empowered, AIDS-free, Mentored and Safe (DREAMS) initiative in 15 countries, including Malawi, to target the structural and behavioral factors that increase AGYW risk of acquiring HIV [45]. A target of the DREAMS initiative is to reduce the likelihood of early marriage in AGYW; however, among AGYW who are already married it would be important to mitigate high-risk behavior within their partnerships, such as the current programming around condom promotion and provisions among AGYW and their partners [46]. 

Our study suggests that HIV prevalence was higher among AGYW in age-mixing sex partnerships compared to those in age-concordant sex partnerships, aligning with studies conducted in South Africa, Kenya, Zimbabwe, and Uganda [27, 28]. This consistent finding lends credence to the generalizability of our study findings to AGYW with primary sexual partners in the broad sub-Saharan African context. Other studies of age-mixing sex partnerships have revealed inconsistent associations with HIV prevalence, but these studies were conducted in more rural settings, where access to resources is limited but also risky behavior may be lower [47]. Other studies have found that settings where AGYW have frequent interactions with researchers may have been informed of the risks of engaging in sexual relationships with male partners or included AGYW willing to enroll in a randomized clinical trial and use highly effective forms of contraception lowering their overall perceived and actual risk of HIV [19, 26]. Despite these differences, our results were consistent with findings that showed no association between age-mixing sexual partnerships and HIV among two-thirds of our analysis population when we did not consider endorsement of inequitable gender norms. This finding suggests that there might be a higher risk among a minority of AGYW who endorse inequitable gender norms and not all AGYW.

Previous studies in Malawi have also assessed the association between age-mixing sex partnerships and HIV infection among this maturing population [10, 29, 48]. Our results were consistent with a social network study in Likoma Island, Malawi, showing that the risk of being HIV positive in females varied by the age difference with their partners [10]. However, our study found the greatest risk of HIV was among AGYW in intergenerational relationship but in their study as age difference increased between AGYW and their male partner, HIV risk decreased. Still, this relationship may be underestimated as another study conducted in Likoma Island suggests that survey reports of partner age were significantly underestimated, likely missing the true extent of HIV risk associated with age discordance in this population [29]. Given the unique context of Malawi, this population-based study adds to the body of literature; however, additional research is needed in this region.

Although many studies have acknowledged the role of inequitable gender norms as underpinning the vulnerabilities of AGYW in these relationships, no previous studies had quantitatively assessed its role as a modifier to the relationship. Our results support our hypothesis that inequitable gender norms have a strong modifying effect on the association between age-mixing patterns and HIV infection among AGYW in Malawi. Although we did not find differences in endorsement of specific inequitable gender norms by partnership status, this may be due to the prevailing and ubiquitous social and cultural gender norms inherent to Malawi and other countries [28]. We also found a large proportion of our sample reported not knowing the HIV status of their primary sexual partner, further highlighting the potential power dynamics that may exacerbate status non-disclosure between partners. Most notable is the stark increased odds of HIV among AGYW who endorse inequitable gender norms in age-mixing partnerships and how the odds increases with age of their partner. This compounded relationship points to a subset of AGYW at heightened risk and necessitates future research into the complexities of these AGYW.

Studies have assessed the impact of gender norms and associations with HIV risk; however, this analysis is one of the first that highlights inequitable gender norms in this population as a potential point of intervention for the risk associated with age-mixing partnerships [49, 50]. Interventions have focused on women’s vulnerabilities to try to prevent them from engaging in age-mixing relationships and intimate partner violence (e.g., SASA!) but those strategies have had mixed effectiveness and may not consider the complex motivators and perceived benefits of participating in these partnerships [51, 52]. For example, a qualitative study conducted in Tanzania and Uganda showed that despite the acknowledged increased risk of HIV in age-mixing relationships learned from these programs, AGYW receive financial benefits, emotional support, and meet social expectations through these relationships [53]. A new intervention, Reaching Married Adolescents in Niger, is designed to promote equitable gender norms and lower engagement in risky behavior between AGYW and their husbands, and reduce intimate partner violence [54]. Further research should be done in the adaptation and scale-up of this intervention and others that consider the complexity of these relationships in the broader sub-Saharan African setting. The results from our study, and a growing body of intervention research, suggests a need to refocus interventions to address the structural constraints AGYW face, and for those constraints to be integrated into DREAMS initiatives, as well as comprehensive social support and sexual reproductive health services.

It is important to note we cannot infer causality since this is a cross-sectional analysis. However, our findings are consistent with prior studies from other countries that have confirmed this relationship [20, 25]. In addition, since data collection occurred through face-to-face interviews, responses may have been subject to social desirability bias leading to underestimation of risk behaviors. Although this is possible, this study collected a broad spectrum of behavioral risk factors, as well as partner characteristics, to obtain a comprehensive risk profile of the participants and there were significant differences in risk behaviors even if they were underestimated, lending to its strength. Additionally, about 25% (1,108/4,448) of the AGYW who were potentially eligible for inclusion in this analysis but did not have a primary partner or know the age of their primary partner, limiting generalizability. The behavioral profile of this sub-group indicated they were less likely to report higher risk sexual behaviors reducing likelihood of HIV infection; thus, the absence of those individuals are likely biasing our current findings away from the null. However, given the findings of the study conducted in Malawi that found consistent underestimation of partner’s age, our current findings could also be an underestimation of the impact of age-mixing partnerships and HIV [29]. Other considerations of unknown age of partner may be due to a lack of seriousness of the relationship; however, evidence suggest that among AGYW engaging in riskier behavior in their relationship also describe those relationships as more committed [55]. Lastly, the relatively few AGYW who reported intergenerational sex partnerships impacted the precision of our assessments. Still, the nationally representative sampling of this study increases the generalizability to Malawi and other countries with similar HIV epidemics.

## Conclusion

Our findings suggest that age-disparate and intergenerational sex partnerships are risk factors for HIV among AGYW in primary sex partnerships in Malawi. As there continues to be an urgent need for interventions to reduce HIV among AGYW, it is important to acknowledge the role of gender norms and the potential benefits many AGYW may get from engaging in these relationships when developing and implementing interventions. Further research is needed to inform points of interventions that are centered around successfully navigating these relationships, when necessary, while increasing education and economic independence, as well as gender- and self-advocacy.

### Electronic supplementary material

Below is the link to the electronic supplementary material.

Supplementary Material 1

## Data Availability

All Population-based HIV Impact Assessment Project data is publicly available upon request at PHIA data repository: https://phia-data.icap.columbia.edu/. Reasonable requests can be made to access the data analyzed in this particular study from the corresponding author.

## Abbreviations

AGYW: Adolescent Girls and Young Women
GEM: Gender Equitable Men
HIV: Human Immunodeficiency Virus
IRB: Institutional Review Board
LAg: Limiting Antigen
MPHIA: Malawi Population-based HIV Impact Assessment
UNAIDS: Joint United Nations Programme on HIV/AIDS
