# Supplementary material for: Age-disparate and intergenerational sex partnerships and HIV: the role of gender norms among adolescent girls and young women in Malawi
Source: BMC Public Health. 2024 Feb 22;24:575. doi: 10.1186/s12889-024-17868-5 (PMC10885496; doi:10.1186/s12889-024-17868-5)
Supplement: Supplementary file 1 — Supplementary Material 1 [file 12889_2024_17868_MOESM1_ESM.docx]

**Supplemental Material**

**Figure 1. Percentage of Participants who Endorsed Inequitable Gender Norms by Age-mixing Partnership Status**
